# Implementing the skilled birth attendance strategy in Uganda: a policy analysis

**DOI:** 10.1186/s12913-019-4503-5

**Published:** 2019-09-10

**Authors:** Susan Munabi-Babigumira, Harriet Nabudere, Delius Asiimwe, Atle Fretheim, Kristin Sandberg

**Affiliations:** 1 Global Health Unit, Division for Health Services, Norwegian Institute of Public Health, P.O. Box 4404, Nydalen, N-0403 Oslo, Norway; 2 University of Oslo, P.O. Box 1130, Blindern, 0318 Oslo, Norway; 3 Uganda National Health Research Organization, Plot 2, Berkeley Lane, P.O. Box 465, Entebbe, Uganda; 4 Kabano Research and Development Centre, Plot 487, Kyengera, Masanda Zone, P.O Box 35906, Kampala, Uganda; 5 Fridtjof Nansens Institute, P.O.Box 326, 1326 Lysaker, Norway

**Keywords:** Skilled birth attendance, Maternal health, Health system, Policy implementation, Policy analysis

## Abstract

**Background:**

Uganda, a low resource country, implemented the skilled attendance at birth strategy, to meet a key target of the 5th Millenium Development Goal (MDG), 75% reduction in maternal mortality ratio. Maternal mortality rates remained high, despite the improvement in facility delivery rates. In this paper, we analyse the strategies implemented and bottlenecks experienced as Uganda’s skilled birth attendance policy was rolled out. These experiences provide important lessons for decision makers as they implement policies to further improve maternity care.

**Methods:**

This is a case study of the implementation process, involving a document review and in-depth interviews among key informants selected from the Ministry of Health, Professional Organisations, Ugandan Parliament, the Health Service Commission, the private not-for-profit sector, non-government organisations, and District Health Officers. The Walt and Gilson health policy triangle guided data collection and analysis.

**Results:**

The skilled birth attendance policy was an important priority on Uganda’s maternal health agenda and received strong political commitment, and support from development partners and national stakeholders. Considerable effort was devoted to implementation of this policy through strategies to increase the availability of skilled health workers for instance through expanded midwifery training, and creation of the comprehensive nurse midwife cadre. In addition, access to emergency obstetric care improved to some extent as the physical infrastructure expanded, and distribution of medicines and supplies improved. However, health worker recruitment was slow in part due to the restrictive staff norms that were remnants of previous policies. Despite considerable resources allocated to creating the comprehensive nurse midwife cadre, this resulted in nurses that lacked midwifery skills, while the training of specialised midwives reduced. The rate of expansion of the physical infrastructure outpaced the available human resources, equipment, blood infrastructure, and several health facilities were not fully functional.

**Conclusion:**

Uganda’s skilled birth attendance policy aimed to increase access to obstetric care, but recruitment of human resources, and infrastructural capacity to provide good quality care remain a challenge. This study highlights the complex issues and unexpected consequences of policy implementation. Further evaluation of this policy is needed as decision-makers develop strategies to improve access to skilled care at birth.

**Electronic supplementary material:**

The online version of this article (10.1186/s12913-019-4503-5) contains supplementary material, which is available to authorized users.

## Background

Uganda, a low resource country, implemented skilled attendance at birth strategy, to meet a key target of the 5th Millenium Development Goal (MDG), 75% reduction in maternal mortality ratio. Skilled birth attendance in Uganda increased from 35% in the 1990s [1], to 42% in 2006 [2] and 74% by 2016 [3], falling a little short of the 90% target [4]. Maternal mortality rates were reduced from 524 per 100,000 live births in the period 2000–2001 to 368 per 100,000 live births by 2016 [3], far from the stated goal of 131 per 100,000 live births [5]. In this paper, we analyse the strategies implemented and bottlenecks experienced, as perceived by mid-level national policymakers and district managers, when Uganda rolled out the skilled birth attendance policy.

Skilled birth attendance is the process by which women are provided with adequate care during labour, delivery and the postpartum period [6]. Such attendance requires both a skilled attendant and an enabling environment, characterised by supportive regulation, policies and infrastructure, communication, referral, and the necessary logistics and supplies [7]. The World Health Organization’s definition is clear in specifying that skilled attendants must be trained, accredited and skilled health professionals (such as midwives, doctors, or nurses) [8].

However, in several low- and middle-income countries, there is great variation in the cadre, the competence, and scope of practice of birth attendants [9–11]. Not all birth attendants at health facilities have the necessary skills to conduct deliveries. When countries in these settings report the proportion of health facility deliveries as a proxy for skilled birth attendance, this may underestimate the actual number of mothers that receive skilled care. In addition, the content and environmental conditions for provision of obstetric care at health facilities vary widely by context, reflecting the status of the health system.

The complex nature of health systems and skilled birth attendance make an understanding of implementation processes important, especially for policymakers and managers seeking to further improve maternity care [12]. In this paper, the term ‘implementation’ refers to the actions that are taken when carrying out policy decisions, such as the decision to increase skilled birth attendance [13].

### Strategies to improve access to skilled birth attendance in low-and middle income countries (LMIC)

The Countdown to 2015 for Maternal, Newborn and child survival group that monitored progress towards the MDG targets, reported 65% median coverage of skilled attendance at delivery from 66 countries with the highest maternal mortality rates [14, 15]. These countries implemented a wide range of strategies to improve access to emergency obstetric care provided by skilled birth attendants. For instance, some countries implemented community-based strategies to provide obstetric services closer to families, for example through community midwives in Indonesia [16]. Others established birthing centres or maternity waiting homes, for example in Zimbabwe or Malawi [17]. Several countries that lacked sufficient human resources e.g. Ethiopia and Bangladesh, used community health workers to deliver tasks that skilled providers would otherwise have delivered. India implemented private-public partnerships to deliver care in rural communities [18]. Others opted for skills training of health providers and expansion of health services infrastructure to increase access to basic and comprehensive emergency obstetric care [19, 20]. These examples illustrate the variety of approaches adopted by countries, each with its own unique context.

### Uganda context

The implementation of the skilled birth attendance policy in Uganda was shaped by the wider reforms in Uganda’s health care and public sectors. In the early 1990s Uganda implemented public sector reforms to improve the efficiency of the public service, as part of the World Bank’s Structural Adjustment Programs. Health services were downsized and decentralised, and authority for health policy implementation was transferred from the Ministry of Health to district authorities [21].

Further, Health Sub-Districts (HSDs) were created as new administrative levels below the district, and their management located at Level IV Health Centres or District Hospitals (see Table 1 for an overview of the health system in Uganda). Health centres were renovated, and new facilities, such as maternity wards and surgical theatres, were constructed. Medical doctors and anaesthetists were included among the staff at Level IV Health Centres to provide more specialised services, such as comprehensive emergency obstetric care. As part of the changes, Level III Health Centres were assigned to provide inpatient maternal care and Level II Health Centres provided outpatient maternal care.

**Table 1:** Overview of the health system in Uganda

Health Facility	Roles
Regional and National Referral hospitals	Provide ccomprehensive specialist services, conduct research and training of health workers. Provide all other services as district level hospital. Target population two million people.
District (General) Hospitals	Provide curative, preventive, maternity, outpatient and inpatient services. Provide blood transfusion, medical imaging and laboratory services. Provide in service training, support community based programs through consultation and research. Target population 500,000 people
Health Centre IV (HCIV)	Provide curative, preventive, maternity, outpatient and inpatient services. Supervise, coordinate and plan for health centre levels III and II. Target population 100,000 people.
Health centre III (HCIII)	Provide basic preventive, promotive and curative care as well as basic obstetric care. Support supervision of community and health centre level II. Target population 20,000 people.
Health centre II (HCII)	Provide outpatient care including antenatal care, preventive care and link with the community and village health teams. Target population 5000 people

Adapted from Health Sector and Strategic investment Plan [4]

As part of this decentralisation, districts were expected to increase their financial independence by generating their own revenue to supplement central government funding. However, over time local revenues in many districts have remained unchanged or dwindled [22], and conditional grants from central government, earmarked for the implementation of specific programmes, have increased. Districts therefore continue to depend on central government funding that is limited, and supplement this with donations from civil society organisations. The National Health Accounts 2016 report published by Ministry of Health, indicated that between 2012 and 2014, Uganda’s expenditure on the health sector was 7–8% of gross domestic product, and was less than the 15% target agreed by African Union countries in the Abuja Declaration of 2001. In the same period, about 14% of expenditure on health was for reproductive health conditions [23].

Against this background, we sought to answer two key questions: i) what strategies were implemented in Uganda to scale up skilled birth attendance? and ii) what can we learn from the implementation of these strategies that could contribute to our understanding of the process required to attain skilled birth attendance targets?

## Methods

### The study site

We conducted our study in Kampala, Uganda’s capital city, where national level policy makers and key actors in policy formulation, including the Ministry of Health that is responsible for coordinating all health policies, are based. The other study areas were Mpigi and Rukungiri districts. We purposively selected these to represent the district level, which is responsible for policy implementation. Mpigi district is in central Uganda, 38 km west of Kampala, while Rukungiri district is in Western Uganda, 373 km southwest of Kampala.

### Study design

We utilised a case study approach to investigate Uganda’s implementation of the skilled birth attendance policy as perceived by mid-level national policy makers and district managers. The case study methodology is particularly well suited to analyse policy implementation, which is deeply embedded in the local context [24]; and to study the complex relationships between actors, and the influence of these relationships on implementation over time [25].

### Conceptual framework

The Walt and Gilson health policy triangle [26], which focuses on an examination of the context, key actors and implementation activities, guided the development of data collection tools and our analytical approach. We traced the shifting interpretations and assumptions about who skilled birth attendants are, and what the implementation of the skilled birth attendance policy has entailed at the national and district levels over time. In addition, we utilised a principal agent perspective which views gaps in implementation as a result of the way governments usually operate, with policy makers delegating functions to other officials or ‘agents’ that are only indirectly under their control and therefore difficult to monitor. Typically, these agents make their own decisions on how they operate on behalf of the ‘principal’, which affects how a policy is implemented [27].

### Data collection

#### Document review

We conducted internet searches and snowballed our search of Ugandan policy documents, as well as documents on global and African regional maternal and reproductive health. For example, we searched the Ministry of Health Uganda website, the websites of key organisations related to maternal health such as United Nations Population Fund (UNFPA), and hand searched selected documents in the Ministry of Health Uganda library. We analysed relevant documents to clarify the ‘skilled birth attendant’ concept within the local Ugandan context, and the strategies (and outcomes of these strategies), the actors, and the key events that played a role in providing skilled birth attendance in Uganda. Information obtained from the document review guided the formative phase of this study and supplemented the data during analysis.

#### Key informant interviews

We purposefully selected key informants from a preliminary list of key actors identified from the document review. We selected key informants that had a role in developing and/or implementing Uganda’s maternal health policy, and had served in their organisation for at least 2 years. In addition, we made a list of our assumptions about the implementation of maternal health policy in Uganda, and used some of these to guide the selection of the informants (see Additional file 1). We assumed, for example, that policy implementation by the private and not-for-profit sector would be different from the public sector and selected informants from both sectors. Two of the authors (SMB and HN) who previously worked as clinicians in Uganda, and are currently health systems researchers, conducted the interviews. The questions focussed on the content and emergence of the skilled birth attendance policy in Uganda and the strategies implemented (see Additional file 2). We conducted interviews in English, for 1–1.5 h, at the workplaces of the key informants, between June and August 2014. Due to the busy schedule of the key informants, prolonged engagement and follow-up interviews were not possible. All the interviews were audio recorded by a research assistant and a moderator (either SMB or HN) took field notes. The moderators probed for information on new ideas arising during the interview or from previous interviews, until no new information arose from the subsequent interviews.

### Data analysis

Research assistants transcribed all the audio recordings. One author (SMB) crosschecked the transcripts against the audio records to ensure accuracy. We used a thematic analysis approach when analysing the data [28]. Two authors, working independently, read and reread the transcripts to familiarise themselves with the data and identify emerging themes. Three authors (SMB, HN, and DA) discussed the emerging themes to identify linkages and define the boundaries of the term ‘skilled birth attendant’ in the Uganda context, how the policy evolved, the strategies implemented, and any bottlenecks experienced during the policy implementation. As part of the analysis, we triangulated the interview data with information obtained from the policy documents to clarify concepts.

## Results

Eighteen semi-structured interviews were conducted among key informants selected from the Ugandan Ministry of Health, Professional Organisations e.g. Nurses and Midwives Council, members of the Ugandan Parliament, the Health Service Commission, the private not-for-profit sector, civil society organisations, and District Health Officers (see Table 2 for respondent characteristics). Apart from two, all the other key informants were healthcare professionals. Six of the respondents had held a position in their respective organisations for more than 10 years and provided a historical perspective of the country’s skilled birth attendance policy.

**Table 2:** Respondent characteristics

Category of Organisation	Organisation or department/Number of participants	Years of Service in current position
Ministry of Health	Reproductive Health Division (2)	> 10/11 years
	Human Resources Division (1)	4
	Quality Assurance Department (1)	5
Autonomous/Professional Organisations	Health Services Commission (1)	2
	Association of Obstetrics Gynecology (1)	17
	Uganda Nurses and Midwives Council (1)	5
	Uganda Private Midwives Association (3)	2/3/3
Private Not for Profit Organisation	Uganda Protestant Medical Bureau (1)	2
Multinational partners	World Bank (1)	14
	World Health Organisation (1)	17
	United Nations Population Fund(1)	8
Non-Governmental Organisations	Reproductive Health Uganda (1)	4
	Uganda National Health Consumers Organisation (1)	6
	White Ribbon Alliance(1)	5
Other Interest groups	National Association of Women Ministers and Members of Parliament Uganda (1)	3
District Health Officers	Mpigi District (1)	19
	Rukungiri District (1)	3

The rest of this section is organised according to the themes that emerged from the analysis of documents and key informant interviews. The first part describes how the policy emerged, the content of the policy, the skilled birth attendant, the key actors and their roles in developing and implementing Uganda’s skilled birth attendance policy. The second part describes the activities and bottlenecks experienced during policy implementation.

### How the skilled birth attendance policy emerged in Uganda

**Fig. 1:**
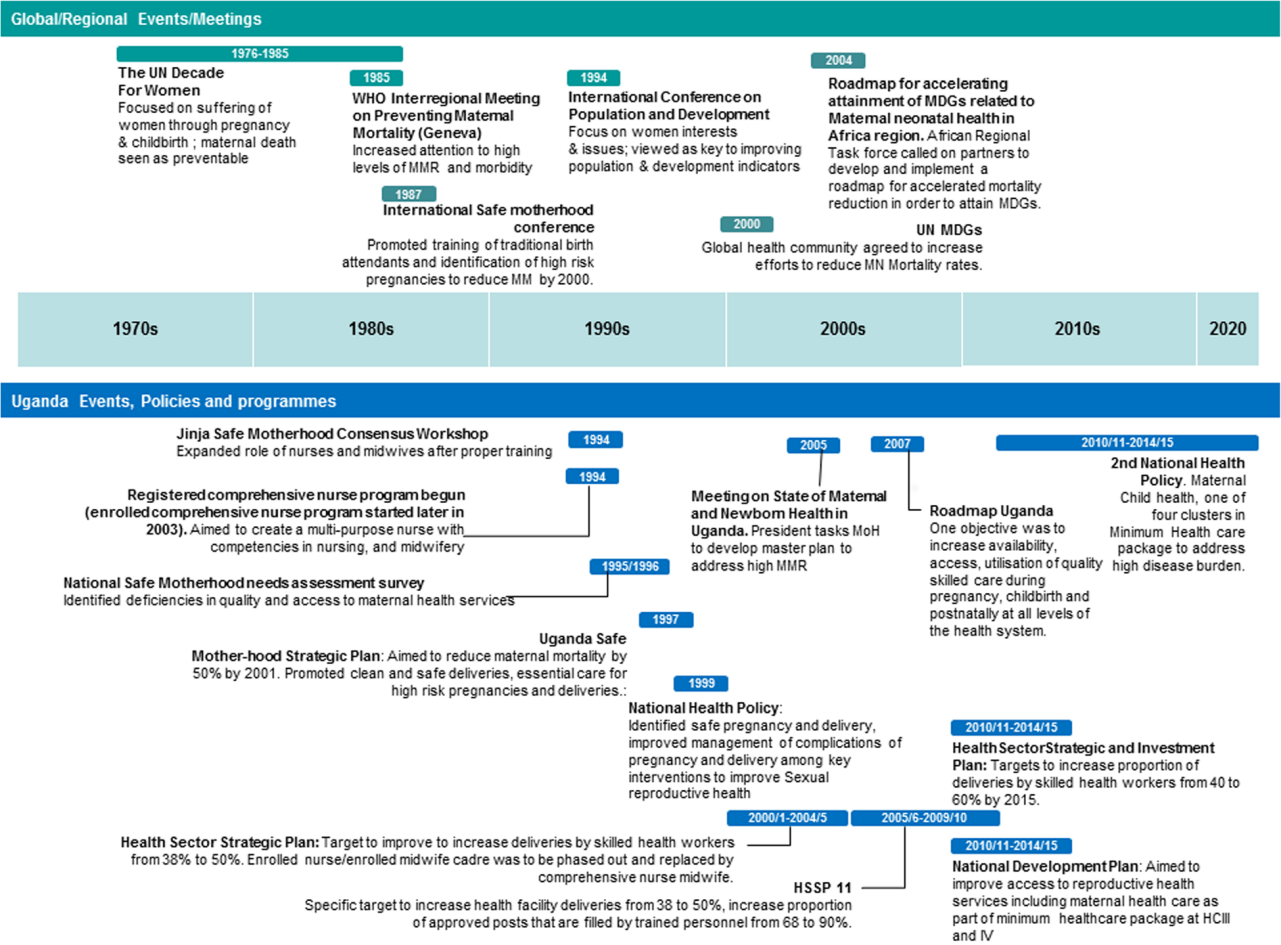
Timeline of Global/Regional/Uganda events, meetings, policies and programs. Legend: Green: Year in which key events and meetings were held at global or regional level. Blue: Year in which key events were held, or policies developed or programmes rolled out in Uganda

International, regional and national events factors pushed the skilled birth attendance policy onto Uganda’s health agenda (see Fig. 1: Timeline of Global/Regional/Uganda events, policies and programs). In particular, the Safe Motherhood movement of the late 1980s brought global attention to the high rate of maternal mortality and the need to improve maternal health. At the 1987 International Conference on Safe Motherhood held in Nairobi, Kenya, countries were tasked with defining country-specific problems and identifying solutions. As a result, Uganda developed the Uganda Safe Motherhood Strategic Plan 1997–1999 [29] which included training of health workers and Traditional Birth Attendants (TBAs) to improve their knowledge and skills for clean and safe deliveries.

By the late 1980s and early 1990s, reviews of Uganda’s Safe Motherhood programme showed that maternal mortality rates had not reduced. Policymakers recognised that TBAs could not manage complications that required access to emergency care and the policy focus shifted to increasing access to skilled personnel.

Globally, a new policy window opened in 2000 when the United Nations adopted the 5th Millennium Development Goals (MDGs) to “improve maternal health”. The target set was to “reduce by three quarters, between 1990 and 2015, the maternal mortality ratio”, using the proportion of births attended by skilled health personnel as an indicator. This helped to focus the attention of governments on skilled birth attendance as a key strategy to curb high maternal mortality rates. Uganda’s first Health Sector Strategic Plan 2000/1–2004/2005 reflected the country’s adoption of this strategy on a policy level.

Regional meetings in Africa have kept skilled birth attendance on the agenda since the United Nations Millennium Summit in 2000. At the African Regional Task Force meeting in 2003, for example, the global health community urged partners to develop and implement roadmaps for accelerating reductions in maternal mortality. Policy stakeholders from Uganda participated in this meeting and subsequently developed the Roadmap for Uganda [30].

Apart from international and regional influences, national factors have also helped keep the skilled birth attendance policy on the agenda in Uganda. For example, following a 2005 high-level plenary meeting of the United Nations General Assembly in New York, the President of Uganda tasked the Ministry of Health to develop a plan to address the country’s high maternal mortality rates. The poor state of the health system has been a problematic political issue during presidential elections, and the media and civil society organisations have continued to publicise the poor state of Uganda’s health services, particularly in maternal and child health [31–33]. Skilled birth attendance has remained an important priority as reflected in several benchmarking studies [34, 35] and Uganda policy documents [4, 36].

### Content of the policy

In their first Health Sector Strategic Plan (2000/1–2004/5), the Ministry of Health of Uganda proposed to scale up skilled birth attendance from 38 to 50% [37]. Although our document review showed that this plan contains the earliest reference to a skilled birth attendance policy in Uganda, the policy was not entirely new. Activities to improve access to maternal health (such as the refurbishment of health facilities to provide emergency obstetric care) had already been included in Uganda’s Safe Motherhood Initiative [29]. Additional activities specified in the first Health Sector Strategic Plan were training to improve the performance of health workers; provision of medicines, supplies and equipment; expanding emergency obstetric services, and strengthening the efficiency of the referral process. When asked to describe Uganda’s skilled birth attendance policy, respondents described a policy focus promoting health facility deliveries. Many reported that the policy encouraged mothers to deliver at health facilities under the care of skilled health personnel; and required the right personnel, equipment, and environment; at the right level within the health system (see Table 1).

### Who is a ‘skilled birth attendant’ in Uganda?

There was consensus among respondents that obstetricians and gynaecologists, medical doctors, nurses and midwives trained and certified in midwifery and/or the care of pregnant women, were considered skilled attendants within the Uganda context. Respondents also discussed other obstetric care providers, for example comprehensive nurse midwives, clinical officers and nursing assistants.

*Comprehensive Nurse Midwives*: Although Comprehensive Nurse Midwives (CNM) are certified health professionals and, by definition, are skilled birth attendants, in practice many lack skills in midwifery and are often recruited to nursing positions. This cadre was introduced in Uganda in the mid-1990s (see further details in section on Human resource strategies).

*Clinical Officers*: Respondents expressed divergent views about clinical officers’ competence in midwifery. One respondent was unsure, and another suggested that clinical officers receive midwifery training, and are skilled birth attendants. However, in practice, clinical officers rarely conduct deliveries due to lack of midwifery training. Uganda had recently included midwifery as part of clinical officers’ pre-service training curriculum (30).

*Nursing assistants*: This cadre is not authorised to conduct deliveries but provides support to health workers. Nursing assistants have attended a 3-month, certified course without a formal curriculum, and have acquired their skills on the job. Over time, due to a combination of human resource shortages and a lack of supervision and regulation, the role of nursing assistants has expanded to include conducting deliveries at health facilities. At present, the Ministry of Health has stopped recruiting nursing assistants and those already recruited into the health system, will attend certified training courses or continue to support health workers until they retire. Some respondents feared that phasing out nursing assistants without providing replacements could worsen the human resource situation in health facilities.

### Key actors and their roles

Table 3 (see Table 3: Key actors and their roles in health policy development and implementation**)** summarises the key actors and their roles in the development and implementation of the skilled birth attendance policy, and is based on our document review and interviews with the key informants**.** At the national level, the Reproductive Health Division of the Ministry of Health has the overall responsibility for maternal health services. The Ministry of Education supports the Ministry of Health with infrastructure for the training of health workers. Several multilateral and bilateral organisations provide technical and financial support for maternal health.

**Table 3:** Key actors and their roles in health policy development and implementation

Actors	Roles
National level	
Reproductive Health Unit, Ministry of Health (MOH)	The Reproductive Health Unit develops policy, provides coordination and guidance, technical support and supervision of the districts for maternal health.
Ministry of Education and Sports	Responsible for pre-service training of health workers, licensing and regulation of training schools
Ministry of Public Service	Recruits and deploys personnel into civil service, defines standards, maintains the payroll, determines incentives including for health workers.
Ministry of Finance, Planning and Economic development	Sources for and allocates finances according to priorities set by the government
National Association of Women Ministers and Parliamentarians, Parliament of Uganda	Influences policy formulation, reviews and approves the government budget. Advocates for maternal child health in fora with the President, Parliament.
Health Professional Councils e.g. Uganda Nurses and Midwives Council and Uganda Medical and Dental Practitioners Council.	License and regulate the practice of health workers and health units, ensures that training schools comply with standards of medical practice. Sets and reinforces standards for professional qualification, health worker performance, patient safety and ethical practice.
Health Professional Associations e.g. Association of Obstetricians Gynecologists, Uganda Nurses and Midwives Association.	Advocate on behalf of their members for professional welfare and standards.
Health Service Commission	Reviews terms and conditions for service, establishes code of conduct of government health workers.
Private not for profit organisations	
Religious Bureaus: Uganda Protestant Medical Bureau, Uganda Muslim Medical Bureau, Uganda Catholic Medical Bureau and Uganda Orthodox Medical Bureau	Contribute to policy formulation at National Level, owns and supervises almost 50% of health facilities that provide health services at national, district and lower levels.
Bilateral/ Multilateral Partners	
The World Bank (Health systems strengthening project)	Funds infrastructural development e.g. renovation and equipment for facilities, strengthening referral systems, improving maintenance.
United Nations Population Fund (UNFPA)	Provides technical support, equipment and supplies for referral and district hospitals. Sponsors training of midwives from hard to reach areas (bonding for 3 years). Provides EMONC training in eight districts. Supports training colleges and the Uganda Nurses and Midwives Council.
World Health Organisation	Provides technical assistance to MOH, funds for needs assessments
United Nations Children’s Fund (UNICEF)	Provided scholarships for 5 years to train 90 health workers in Karamoja region (bonding for 3 years).
United States Agency for International development (USAID), Baylor Project	Scholarships to train, recruit and deploy 500 midwives for five districts in south- west, and northern Uganda. Provides technical assistance for policy formulation, support supervision. Equip laboratories
United Nations Agencies and United Kingdom’s Department for International Development (DFID/UKAID)	Training of midwives through bursaries for Karamoja and northern districts. Funds regional workshops to address maternal health and quality of Care.
European Union	Funds for human resources development
Danish International Development Agency (DANIDA)	Funds for the health-training bursary fund. Funded rehabilitation of training schools, maintenance of equipment.
Embassy of Belgium	Scholarships for an average of 60 new students from hard to reach areas every year.
Civil Society Organisations	
E.g. White Ribbons Alliance, Reproductive Health Uganda, Family Health international	Advocate for accountability and improved maternal service delivery, conduct studies and commission reports
Uganda National Health Consumers Association	Advocate for providers and consumers for quality health care. Advocate for community participation and accountability.
DISTRICT LEVEL	
District Health Officers and Health Team	Implement health policy on behalf of MOH, supervise and manage health personnel.
District Service commission	Recruit, discipline and set conditions of service for district health staff on behalf of Health Services Commission

New interest groups, such as the National Association of Women Ministers and Members of Parliament (NAWMP) have also emerged. NAWMP was formed in 2007 to advocate within the Ugandan parliament on reproductive, maternal and child health issues. In 2010, NAWMP together with the White Ribbons Alliance and other Civil Society Organisations, lobbied for a 130 million USD loan from the World Bank for reproductive health commodities, to renovate hospitals and to purchase ambulances. In addition, NAWMP has advocated for funds to recruit midwives and doctors at Level IV Health Centres. Apart from advocacy, civil society groups have also conducted studies and commissioned reports that have held the Ugandan government and parliament to account, and lobbied for improvements to maternal child health services.

Other actors, such professional organisations, provide technical support to the Ministry of Health through participation in advisory groups, and their members employed in the health service. District level actors implement health policy, and plan, coordinate, and manage health services on behalf of the Ministry of Health.

### Implementation of the skilled birth attendance policy

In the previous section, we described the skilled birth attendance policy and its emergence in Uganda. In the next section, we will discuss the activities and bottlenecks experienced during the implementation of this policy. We categorise and discuss these under ‘Human resource strategies’ and the ‘work environment’. We present selected quotes from respondents in the text. Additional illustrative quotes are provided in Table 4.

**Table 4:** Illustrative quotes on strategies to improve human resources and work environment

Training *‘The ‘life saving skills’ was the first technical programme that was run in the country …*. *they conducted a number of trainings [to] update the skills of midwives and doctors’* (Officer, Autonomous Organisation). *‘Initially the clinical officers did not have a strong obstetrics midwifery component in their curriculum, and this had to be addressed … ..The current discussion and action [is] to review the midwifery curriculum and update it … to ensure that we produce competent midwives’ (*Officer, Multinational partner). *‘United Nations Population Fund has also [been] supporting midwives to go to school and then they bond them in those districts’* (Officer, Professional Organisation). *‘Some time back government took a strategy of comprehensive nurses but different people hold different views regarding that. The idea was to have one skilled person in nursing, midwifery and a bit clinical. I think that did not work. I think the general concern is that many of those comprehensive nurses do not have sufficient skills in midwifery’* (Officer, Multinational partner). *‘The number of midwives has been dwindling because between 2008 and 2010 there were no government schools training midwives. All government schools shifted to training comprehensive nurses both enrolled and registered. That was a disaster. So when we tried to recruit there were no midwives. The only schools that continued training midwives were [four] Private not for profit hospitals’* (Officer, Autonomous Organisation).	
Recruitment and deployment *‘Many midwives have been trained but government is not employing them because government is following outdated staffing norms. You may have 5000 midwives trained, but if staffing norms say recruit 300, only 300 will be recruited’* (Officer, Autonomous Organisation). *‘You see recruitment depends on the wage bill when the wage bill is limited then the district cannot recruit … Also xxx (an NGO) has also supported us in recruitment of midwives mean while we are waiting for the general government recruitment’* (District Officer). *‘I think government has tried to give us more money for recruitment of the right cadres, improvement of infrastructure to house those recruited’* (Officer, MOH). *‘You find those who are trained are actually not recruited because of the markup that government has placed on its ability to pay for the civil service number of personnel. So you find that although the training of cadres of doctors and midwives somehow increased, the recruitment has been literally very static’* (Officer, Professional Organisation). *‘For us xxx (institution) we would have loved to have 100% recruitment but then government would say it doesn’t have the money for salaries and other things. …*. W*e have agreed that we continuously advocate to ensure that the gaps in staffing are filled up’ (Officer, Interest Group).*	
Attraction and retention *‘Yes that was mainly done for doctors, salary was increased and they posted to Level IV Health centres. Midwives will also have an increment …*. *there is an increment of 18% for registered nurse and 13–14% for enrolled midwife. But in terms of money, this is very little, I don’t think it* will *offer a lot of attraction, when you put it in real money terms it is very little’* (Officer, Civil society organisation). *‘Some people have tried performance improvement strategies like recognition of best performers but that is in a few isolated districts …*. *Other project-based incentives in districts involve sponsoring those who want to be midwives and after training you tie [bond] them to the district for some time’* (Officer, MOH). *‘Unfortunately, we did not have a strategy deliberately to retain midwives in health centre level II and III throughout the country. We paid them like any other health worker, we made them struggle to look for accommodation like any other health worker. So invariably, we failed to attract and retain midwives in remote areas’* (Officer, Autonomous Organisation).	
Infrastructure *‘It is difficult to know what has actually happened. I think there has been a lot of effort put in by government. The whole concept of level III to IV health centres … refurbishing maternity or units in this country, has been premised on the need to improve access of emergency obstetric care … But … every time people do assessments on emergency obstetric care, the gaps are still there’* (Officer, Multinational partner). *‘Yes a lot has been done in terms of renovation, re-modelling of many health facilities and some hospitals. There has been procurement of ambulances, construction of delivery wards at every sub county where we have level III health centres the government and partners have also procured delivery equipment. … There has been some provision of staff accommodation’* (Officer, MOH). *[*When asked if the plan was to continue expanding the infrastructure*]: ‘I think we want to consolidate by getting the right staffing’* (Officer, MOH). *‘The way to go is to equip level III health centres, improve on the supplies especially [the] delivery equipment [up to] Health centre level II where there are midwives’* (District Officer). *‘Majority of people go to level II health centers because it is again the nearest to the population. So possibly that is what we should be emphasizing … if obstetric services [are available at] 50 miles, and there is a place of 5 miles where I can get a midwife, most people will go [to the nearest unit]’ (*Officer, Professional Organisation).	
Medicines and supplies *‘This has improved a lot. We used to have many stock-outs even for the basics like fansidar for pregnant mothers but these days level II and III health centres are given predetermined basic kits every 2 months. This is more regular than it was. We revise contents regularly and it may not fit everybody but I think it has improved things. Then level IV health centres and hospitals are supposed to estimate what they want and use their budgets. There are still some challenges but I think is now much better’* (Officer, MOH). *‘If you go to many of the facilities they have drugs. So in terms of resources I think that is okay. In terms of clearly defining what drugs are needed and ordering them on time, I think there is still a challenge there because these drugs are supplied within a certain framework of time. So if they get finished it is difficult to replenish because you have to wait for the next supply to come through. So to me, the flexibility is the challenge’* (Officer, MOH). *‘There are many arguments, when you talk to the districts they seem to have [some] problems with it. When you talk to National Medical Stores, they say they are delivering enough. …*. *for accountability purposes I think it is much better, they have decreased a lot of pilferage of resources, but it also has its limitations, at times it was not supplying enough supplies for a unit’* (Officer, Professional Organisation). *‘Some of (the challenges) is poor communication but also the way the districts make the priority list. When they are making the list, they have little money and they do not include [some items]. The way they make the list sometimes is not informed by the needs, other times it is not just a priority for them. I think it is a gender issue. Also they would rather have antibiotics and panadol and let the mothers find what to do. That is part of it but I think secondly, the money the districts get is not enough’*. (Officer, Civil society organisation).	

### Human resource strategies

#### Training

From the 1990s, Uganda’s human resource policy focused on improving the skills and numbers of health workers providing obstetric care. The Ministry of Health in collaboration with the Ugandan Association of Obstetricians and Gynaecologists, and the American college of Nurses and Midwives, developed the Life Saving Skills (LSS) programme, an in-service training programme designed to improve the skills of doctors and midwives in emergency obstetric care. The LSS was scaled-up into a pre-service curriculum for doctors and students of midwifery. Apart from the LSS programme, some doctors, nurses and midwives received training in basic obstetric anaesthesia [38].

From 1994, the Ministry of Health established a new cadre, the Comprehensive Nurse Midwife (CNM), to increase the number of health workers with nursing and midwifery skills, and link communities to the health system. Policy makers and partners viewed the development of the CNM cadre as more economical than training of specialised enrolled/registered nurses or midwives.

The creation of the CNM cadre received mixed reactions in Uganda. Some actors viewed this positively because of human resource shortages. The Ministry of Education found it easier to train CNMs because they required less practical exposure compared to midwives, and stopped offering courses for enrolled/registered nurses and midwives. The private not-for-profit (PNFP) sector group of health care providers, were hesitant about the costs of developing a programme to train CNMs until about 2005 when the European Union and Danish International Aid subsidised the costs of the training programme. The PNFP sub-sector recognised that the skill set of specialised midwives was different from CNMs and continued to train midwives.

Some respondents noted that the CNM strategy had unintended results. From 2011 to 2013, the number of registered CNMs was four to six times higher than the number of midwives registered by the Uganda Nurses and Midwives Council. The impact of the CNM strategy on midwifery was summarized as follows: ‘ … *when we changed the policy (to) training the comprehensive nurses then we had the gap of the traditional trained midwives … it really caused us shortage of skilled midwives. When the comprehensive nurses came on board they were shared to work in clinical areas and general nursing because they had less skills for midwifery. So when it came to fill the vacant positions, midwives were not there in required numbers.’* (Officer, MOH).

An evaluation conducted by the United Nations Population Fund (UNFPA) in 2010, showed that Uganda’s CNM training did not meet the Global Standards for Midwifery Education in part due to insufficient curriculum preparation. Most CNMs have followed a career path into management or nursing positions. The Ministries of Health and Education are yet to agree on the future role of the CNM cadre. The Ministry of Education reintroduced courses for enrolled/registered midwives in all public health training schools in Uganda. PNFP institutions also provide 6-month courses for CNMs seeking to upgrade their midwifery skills.

Other strategies to increase the supply of skilled health workers are revising the clinical officers’ training curriculum in 2002/3 to include a midwifery component; revising the midwifery curriculum in 2013 to ensure compliance with the Global Standards for Midwifery Education; and funding the Uganda Nursing and Midwifery Council to speed up the registration and licensing of midwives [39]. In addition, several development partners have provided scholarships to train midwives from underserved districts (see Table 3). UNFPA, for example, provided bursaries in 2007 to train midwives from districts that were recovering from the effects of war, and an additional eight districts in 2010. Districts lacking eligible candidates received career fares that promoted midwifery in schools.

### Recruitment and deployment

The recruitment and deployment of health workers into the health service has been slow despite considerable efforts to increase supply. Standard health facility-based norms determine the number of health workers at facilities [37]. Several actors have proposed a review of these norms as a way of increasing the number and scope of the health workers recruited [4]. In the interim, recruitment depends on available vacancies and funding e.g. donor funding for specific projects. The situation was explained as follows:

‘*Another challenge in the recruitment was that the wage bill was very small, and the policy was not to fill the vacant positions at facility level but to replace those that have left, died and retired.’* (Officer, MOH).

Occasionally, the Ministry of Health has actively recruited health workers into the health service. For instance, following the creation of health sub-districts, the Ministry of Health recruited doctors and midwives to Level IV Health Centres. In 2012, following advocacy from Civil Society Organisations and the Ugandan Parliament, the government provided additional funds for the targeted recruitment of 10,000 health workers. However, several bottlenecks constrain recruitment of health workers. For example, the staffing norms for health facilities are static, restrictive and unaligned to the needs of Uganda’s growing population and the rising demand for services. This was explained as follows: ‘*Despite the [decision] that we need to increase the numbers, even the positions that government has provided for are not [enough] – even if they are filled 100%, they don’t meet the minimum that is required. For example, we know that in terms of human resources for health, the positions that are filled are about 54% but also those that are provided for are not even 70% [of what is needed]. So that is still a gap.’* (Officer, Private not for profit organisation).

In addition, due to financial limitations, the government and districts are only able to recruit a small number of health workers each year. The recruitment process at the district level is expensive and, in some areas, fails to attract any applicants. Consequently, there is growing demand from actors within the health sector to revert to the national-level recruitment and deployment of health workers.

### Attraction and retention of health workers

In Uganda, pay reforms in the early 1990s increased salaries for employees in the public sector and included provision of a consolidated allowance covering lunch, transport and housing costs [38]. However, the introduction of a 30% pay-as-you-earn tax at the same time minimised these benefits. Since then, salaries for public service personnel have remained unchanged and have led to several strikes by health workers demanding salary increases.

To attract health workers, the Uganda Ministry of Health developed a Motivation and Retention Strategy for Human Resources for Health [40], outlining key strategies to improve retention and recruitment. These included financial incentives, such as salary increases, lunch pay, hardship allowances, and performance-related pay. Non-financial incentives included improvement of accommodation, equipment and supplies to ensure more productive and safer working environments.

However, the Ministry of Health did not include a cost estimate for the Motivation and Retention Strategy and it is yet to be fully implemented. The government of Uganda has provided some incentives to health workers in critically underserved areas: medical doctors recruited at Level IV Health Centres, for example, were offered a monthly salary of 1000 USD (2.5 million Ugandan Shillings (UGX)) in 2012/13) compared to the standard salary of 600 USD (960,000 UGX) for doctors at other hospitals. In addition, the government has increased salaries in hard-to-reach areas by 30%. The government and some districts have also provided non-financial incentives such as accommodation for health workers. Some development partners and civil society organisations have provided performance-based incentives, sponsorship for training, and bonded newly recruited health workers for up to 3 years after training. Apart from scholarships for continuing education, the private not-for-profit sector provide uniforms, gratuities, and refreshments during tea breaks.

Attracting and retaining health workers remains challenging. Some health workers consider the salaries and incentives insufficient to attract them to rural districts. In addition, limited transport, accommodation for families, and schools for the children deter health workers from working in rural areas. Bureaucratic delays in the management of the payroll in some districts has also meant that health workers have received their salaries late. Inadequate supervision and poor living conditions have also contributed to low job satisfaction, absenteeism and high staff turnover [41]. Issues affecting attraction and retention of health workers were summarised as follows: *‘Many times you want to recruit somebody to go and work up country but social amenities like electricity, telephone network and housing are not there: those are some of the things that people [health workers] look at. So many of them will even be posted but within 1 week she is out of there. So that has been a big bottleneck. We try to give the hard to reach incentive for those people who work in rural areas but it is so small; about 30% of their personal salaries. It doesn’t make a lot of sense that they will be working there [in hard to reach areas]. For me that is a very big problem. What would may be make someone stay there [in hard to reach areas] is tripling their salary compared to a person working in an urban area.*’ (Officer, MOH).

### Work environment

The Ministry of Health has implemented several strategies to improve the infrastructure, equipment, and distribution of supplies.

#### Infrastructure

Uganda’s investment in the expansion of health infrastructure dates back to the 1990s when health services were decentralised, and continued with the MDGs. With funding from development partners e.g. the World Bank, USAID’s Baylor Project and UNFPA, Level IV Health Centres were constructed and maternity wards and staff houses built or renovated. In addition, the Uganda government equipped Level III Health Centres, and provided ambulances for hospitals.

Despite the achievements made, the respondents noted that the physical environments of health facilities are not adequate. The quality of care, they suggested, is poor and facility delivery remains unattractive to some women. Level IV and III Health Centres in some districts are not fully functional due to a lack of electricity and other essential equipment. Some theatres are not utilised because they are poorly designed and lack equipment and anaesthetists. Inadequate number of beds, limited space in the labour wards, and unreliable water supplies, also affects the quality of care at some facilities. Housing for midwives is also still inadequate for their families who often reside in urban areas. As a result, health workers often travel to visit their families, and this interrupts 24-h care at health facilities and contributes to absenteeism. Other problems include lack of transport for emergency referrals from rural health centres, and the general lack of infrastructure for blood transfusion in health care settings below the hospital level. Although the Ministry of Health planned to expand physical infrastructure for essential obstetric care down to Level II Health Centres, the implementation has been slow due to lack of equipment and restricted staffing norms. Some key informants suggested that limited non-wage funding for lower level health facilities, and poor management, contribute to the poor functioning of health facilities. In one district, for example, a Level III Health Centre receives 150,000 UGX (50 USD) monthly, and a Level II Health Centre receives 120,000 UGX (40 USD) after bank charges are deducted, for recurrent non-wage expenditure (expenses for medicines and supplies are provided centrally). These funds are insufficient for maintaining health facilities. The challenges from the physical infrastructure and the underlying factors were explained as follows: *‘In our assessment, we found out that a lot still needs to be done e.g. some of the facilities do not have a labor ward, placenta pits, running water. Others did not have a reliable source of power -electricity, e.g. some of those with electricity, have unpaid bills because the primary health care non-wage bill is very little …*. *It cannot meet the cost of running these facilities, e.g. paying for electricity is sometimes a challenge. Where they have a generator, they cannot repair it, because the money is not adequate. Those are some of the infrastructural challenges.’* (Officer, CSO).

*‘The gaps are really not because of major deficits but because of minor issues. It is not the lack of a maternity or the lack of a theatre, but maybe there are no gloves, there is only one midwife, there is no blood, they cannot give intravenous fluids. That is what is compounding the problem. Therefore, when you do an assessment you see that there are gaps, which need to be addressed. Those are more management issues because they reflect management weaknesses which are often in those health facilities.’* (Officer, Development partner).

*‘At my level we are not able to address some of these [in reference to how they are addressing infrastructure and supplies deficiencies] because the money they give us is very little and we have been using it to improve accommodation. In some [Level III Health Centres], we have to construct some houses this financial year but when it comes to repairs and water supply, we cannot do much. The solution is for government to increase funding’.* (District Manager).

The respondents had differing opinions on whether expansion of the infrastructure at health facilities should continue or not. Some respondents suggested continued expansion for populations with limited access to obstetric care, but with efforts concentrated on areas with greatest need. Other respondents disagreed with further infrastructural expansion, but suggested consolidation of the gains achieved so far for example by equipping health units, providing electricity and running water, improving accommodation, and strengthening the referral systems and communication between Level III Health Centres and hospitals.

#### Medicines and other supplies

To improve the distribution of medicines and supplies to health units, the Ministry of Health and the National Medical Stores developed a Pull and Push Strategy. Once every 2-3 months, hospitals and Level IV Health Centres, request medicines and supplies from the National Medical Stores (the Pull Strategy), while Level II and III Health Centres receive a pre-specified consignment (the Push Strategy). Twice a year, the National Medical Stores revise the essential medicines and supplies list to reflect the demand at health centres. ‘Mama kits’ which contain the essential items needed for clean deliveries are also distributed as part of these supplies. In some districts, project-based support e.g. from UNFPA for additional supplies, medicines and equipment has resulted in a considerable increase in deliveries at health facilities.

Respondents expressed mixed opinions about the pull and push strategy. Some respondents described how the regular distribution of medicines and supplies had resulted in fewer stock outs, improved the quality of the medicines available, increased accountability, and minimised financial losses. Others argued that the system was not yet flexible enough to respond to the stock outs that still occur due to poor communication between health facilities and the National Medical Stores, poor forecasting, the rise in demand, and wastage when supplies expire. A few respondents suggested poor forecasting of drugs and supplies was because some health workers lack management skills.

## Discussion

The skilled birth attendance policy was an important priority on Uganda’s maternal health agenda and received strong political commitment, and support from development partners and national stakeholders**.** Considerable effort was devoted to implementation of this policy through strategies to increase the availability of skilled health workers for instance through expanded midwifery training, and creation of the comprehensive nurse midwife cadre. In addition, access to emergency obstetric care improved to some extent, with expansion of the physical infrastructure, and improved distribution of medicines and supplies.

Our study found that despite the effort to train more health workers, strategies to recruit and retain health workers were insufficient to attract and improve human resources at health facilities. This gap in human resources resulted in informal task shifting, as unqualified nursing assistants based at health facilities took on tasks from skilled health workers. Other research has suggested that limited human resources can weaken available care at health facilities by increasing workloads, waiting times, and limit 24-h emergency obstetric care [42]. Whereas, task shifting to other cadres has been suggested by the global community as a strategy to overcome these deficiencies [43], informal task shifting as illustrated in this case study, can result in unskilled providers attending births, who may not be able to detect, manage and/or refer complications when they arise [44].

We also found that despite increased efforts to expand the physical infrastructure, the environment in some health facilities remained poor. The availability of medicine and supplies, water and electricity, blood transfusion infrastructure, transport and communication between health facilities, and accommodation for health workers were particularly challenging. Other research suggests that structural problems such as these, may influence the quality of care health workers are able to provide [45] and affect health worker motivation and retention at their workplace [46]. In addition, structural problems may influence mothers’ perceptions about the quality of available care at health facilities and, in turn influence skilled birth attendance rates [47, 48].

Our case study demonstrated how policy implementation can have unexpected consequences. Uganda’s strategy to create the comprehensive nurse-midwife cadre serves as an example: While the intention was to provide multi-skilled staff, this strategy increased the number of nurses without midwifery skills, and reduced the number of specialised midwives as resources were diverted to creation of this cadre. A study by Amandau et al. [49] similarly found that the introduction of the CNM cadre failed to achieve its intended goals, due in part to the insufficient groundwork in the development of a CNM curriculum and deployment within the Ugandan health system. Another study by Filby et al. suggested that short-term trainings underestimate midwives’ decision-making and responsibility, and short-term training of multipurpose health-workers in particular resulted in poor outcomes for mothers and their babies [50]. The findings from our case study likewise point to the importance of curriculum preparation, training infrastructure, and the time needed to develop a competent and skilled midwifery workforce.

Some scholars have suggested that policy implementation may fail to achieve the intended results if key implementation conditions are not fulfilled. This can occur, for example, when policy makers rely on actors such as district managers, that may lack the necessary skills in health services management [51]. Others have argued that policy implementation may have unexpected effects because of the way target groups and actors at the micro-level may interpret and adapt to policies developed by central actors [52, 53]. A reliance on other agents to implement policy may also lead to unintended outcomes if such agents act at their own judgement to decide what policies to implement [54]. The complex adaptive nature of health systems and the broader environments in which they are located, as well as variations in the local contexts, may further shape the effects of implementing policy decisions [55].

Our findings showed that previous and ongoing policies may have influenced the implementation of the skilled birth attendance policy. In particular, the staffing norms instituted as part of the health service reforms of the 1990s may have restricted recruitment of health workers. Respondents attributed the strict staffing norms to Uganda’s limited expenditure on the health sector, which, like several other African countries [56, 57] is below 10% of the total government budget.

In addition, Uganda’s decentralisation policy has given limited power to district authorities to implement government policy. District Health Officers are ‘agents’ of the Ministry of Health for implementation of health policy [27]. However, most decision making power still lies at the national level and the district authorities have little/no control over wage and non-wage allocations, and have limited autonomy to respond to local priorities. Jeppsson et al. [58] reported similar findings in their comparative study of the decentralisation of the health sector in Uganda and Zambia. They noted that while power was formally devolved to the district level in Uganda, the central governments’ continued control over finances may have contributed to the deterioration of immunisation rates in the late 1990s. Similarly, Atkinson’s study of health reform in Brazil noted that the limitations of decentralisation processes can influence policy implementation. The authors noted that the organisational structure, political culture, and particular management styles of districts can influence the benefits of decentralisation. The level of local autonomy and the degree of responsiveness to local needs, they note, can influence the quality of health care provided [59].

That Uganda attained close to 60% skilled birth attendance rate by 2011 but still did not significantly reduce maternal mortality rates may imply that skilled attendance at delivery was not the only important factor for determining maternal mortality rates. Bangladesh provides an example of a country where skilled birth attendance rate was 32% by 2011, but where they came close to attaining their 5th MDG target after attaining a 5.6% annual reduction in maternal mortality. Arifeen et al. [60] reported that an increase in access to and use of health facilities was an important contributing factor to this success. However, the authors also suggested that a decrease in fertility rates from five to two children per woman in reproductive age, improvements in transportation, communication, and household economy might have contributed to this success. In contrast, Uganda’s fertility rate remains high at 5.4 children per woman [3], and poverty rates are high with 27% of households unable to afford their calorific needs. However, other socioeconomic indicators show some improvement with female literacy rates at 67, and 73% of households own a mobile telephone [61]. Although we did not intend to examine the role of socioeconomic and other demand side factors, these factors are clearly important determinants of skilled birth attendance and maternal health in general.

### Strengths and limitations

Trustworthiness of our study was enhanced by including a wide selection of key informants some of whom had many years of experience with the maternal health programme and provided a historical perspective of the implementation process. We triangulated data from the interviews with data from a document review. The document review enabled us clarify dates and verify the data obtained from the interviews.

We acknowledge some limitations: 1) We base our findings on a few interviews and document review that may not represent all relevant information about implementation of the policy. Additional data on education and skills of the nursing and midwifery cadre providing maternity care would have been useful. 2) This study focusses on a single case of policy implementation. We are therefore cautious not to make inferences about causal relationships 3) Our findings are specific for Uganda at this period in time, and are not likely to be directly applicable to another context.4) We did not include the perceptions of health workers and mothers of the skilled birth attendance policy in this study. End user perceptions on quality of maternity care were published in a separate study [62]. Despite these limitations, this study contributes valuable lessons on policy implementation from the Uganda experience.

### Implications for policy and research

To further improve skilled birth attendance, managers in Uganda need to clarify the competence of health providers conducting deliveries at facilities, and develop strategies to attract, recruit and deploy skilled health workers to areas of greatest need. A revised human resources for health policy that addresses challenges with human resource recruitment and retention is needed. In addition, the policy focus should ensure facilities are not only accessible, but also functional and capable of delivering good quality care for instance by defining a minimum acceptable standard of structural quality expected at each level of the health system. The Ministry of Health should consider establishing mechanisms for evaluation, feedback and learning as part of policy implementation processes. These would provide opportunities for fine-tuning policies during implementation and could prevent well-intended policies from negatively affecting health services. Assessments of how previous policies may affect the implementation of new policies should also be considered, particularly in instances where policy objectives may have changed or be in potential conflict. The piloting of policies in selected districts may also provide good learning opportunities prior to scale-up. An evaluation of policies that are implemented at scale may likewise provide valuable learning opportunities.

## Conclusions

Uganda’s skilled birth attendance policy aimed to increase access to obstetric care through strategies to increase health worker supply, expand the physical infrastructure and improve availability of essential commodities and equipment at health facilities. However, strategies to attract, recruit and retain human resources, as well as ensure fully functional health facilities that are capable of providing good quality care remain a challenge. This study highlights the complex issues that arise from policy implementation, for example the unexpected consequences that could arise when new strategies are implemented, or when new strategies interact with other ongoing policies. In addition, we suggest that managers play an important role in policy implementation. When managers lack skills, resources or decision-making power, local priorities that may be important determinants for shaping the outcome of the policy may not be addressed. We recommend further evaluation of the Ugandan skilled birth attendance policy, as decision makers consider what more needs to be done to improve access to skilled care at birth and maternal health in general.

## Additional file

Additional file 1: List of researchers’ assumptions regarding implementation of Uganda’s skilled birth attendance policy. (DOC 24 kb)

Additional file 2: Interview guide for policy makers and district managers. (DOC 46 kb)

## Data Availability

The data used and/or analysed for this current study are available from the corresponding author on reasonable request.

## Abbreviations

BA: Bachelors of Arts
BSC: Bachelor of Science
CNM: Comprehensive Nurse Midwives
HCII: Health centre II
HCIII: Health centre III
HCIV: Health Centre IV
HSDs: Health Sub-Districts
HSSP II: The second Health Sector Strategic Plan
LSS: Life saving skills
MA: Master of Arts
MD: Medical Doctor
MDG: Millennium development goal
MM: Maternal mortality
MMR: Maternal mortality rates
MOH: Ministry of Health
MPH: Masters Public Health
MSc: Master of Science
NAWMP: National Association of Women Ministers and Members of Parliament
PHD: Doctor of Philosophy
PNFP: private not-for-profit
TBAs: Traditional Birth Attendants
UN: United Nations
UNFPA: United Nations Population Fund
USAID: United States Agency for International Development
USD: United States Dollar
