# Supplementary material for: Implementing the skilled birth attendance strategy in Uganda: a policy analysis
Source: BMC Health Serv Res. 2019 Sep 10;19:655. doi: 10.1186/s12913-019-4503-5 (PMC6734264; doi:10.1186/s12913-019-4503-5)
Supplement: Supplementary file 1 — List of researchers’ assumptions regarding implementation of Uganda’s skilled birth attendance policy. (DOC 24 kb) [file 12913_2019_4503_MOESM1_ESM.doc]

# Additional file 1: List of researchers’ assumptions regarding implementation of Uganda’s skilled birth attendance policy

- Global and regional policy influences how a particular policy rises to the agenda in the country
- There may be differences in perceptions or experiences between national level policy makers and district level implementation. District level may be unable to fully implement a policy as the locus of control remains at the national level.
- Policy implementation by the private not for profit sector may differ from the public sector given their differences in funding, organization and management.
- There may be no prior assessment of on the ground realities before policies are implemented e.g. some facilities that are mandated to provide obstetric care may lack the necessary infrastructure.
- Implementation failure may be largely due to the poor state of the health system over time
