# Supplementary material for: Implementing the skilled birth attendance strategy in Uganda: a policy analysis
Source: BMC Health Serv Res. 2019 Sep 10;19:655. doi: 10.1186/s12913-019-4503-5 (PMC6734264; doi:10.1186/s12913-019-4503-5)
Supplement: Supplementary file 2 — Interview guide for policy makers and district managers. (DOC 46 kb) [file 12913_2019_4503_MOESM2_ESM.doc]

# **Additional File 2:** Interview Guide for Policy makers and District Managers

*Introduce yourself and thank the respondent for agreeing to the interview.*

State your name; a researcher from Makerere University College of Health Sciences, working on the Skilled Birth Attendants project.

**What the study is about:** Skilled attendance at birth is widely recognised as an important strategy that is necessary to reduce the high maternal mortality rates and pregnancy related disabilities in many low-income countries. However, several countries in these settings are yet to achieve the 90% coverage by 2015 target set in the United Nations fifth Millennium Development goal (UN 2011). The need to strengthen health systems in order to attain this and other targets set in the millennium development goals has been emphasised (Freedman 2005, UN 2010).

**Purpose of this study:** To explore government efforts to scale up the proportion of deliveries conducted in health facilities in the Ugandan context and the strategies that have been proposed and/or utilized to recruit, deploy, and retain skilled birth attendants. The lessons learned from this study will be useful for decision makers seeking to strengthen health systems and improve the quality of care for mothers and children in low-income countries.

You are a key stakeholder in the development and implementation of the strategies to scale up deliveries in health facilities, and have been selected to participate in this study. You are kindly requested to provide insights based on your official and personal experience in the development and implementation of the strategies to scale up deliveries in health facilities. This interview will take not more than an hour.

We wish to assure you of utmost **confidentiality** with the information you provide during this interview.

We request your signature to confirm consent to participate in this study. (*Take two copies and leave one with the respondent*).

---------------------------------------------------------------------------------------------------------------------

| ***Respondent characteristics***  Name of Organisation/Institution of Affiliation:  Current Position:  Length of service in this position:  Length of service as a policy maker:  Training relevant to your current position/Professional qualification: |
| --- |
| ***Policy Development***  What is the current policy on health facility deliveries in Uganda?  How did this policy come about or come onto the agenda in Uganda?  ***(Probe for the role of national, regional, global priorities)***  Who were the main actors involved in getting this policy onto the agenda in Uganda? By actors, I mean individuals or organisations that may have influenced the rise of this policy onto the agenda.  What resources did they have at their disposal that may have influenced how this policy got onto the agenda? Resources could include ideas, influence, interest, membership; finances etc. (*Could ask where to find this information*).  To what extent were you involved in the development of this policy? |
| ***Policy Implementation***  Which particular cadre are supposed to conduct health facility deliveries and why? (*Probe*: W*hich health worker cadre qualifies to be a skilled birth attendant in Uganda?)*  Since 1999, Uganda has proposed to increase the health facility deliveries from 38 % (HSSP II) to 65% (HSSIP). What strategies has Ministry of Health implemented to achieve this?  Official and other sources of data show a slow increase in the proportion of health facility deliveries (AHSPR 2012/2013). In your opinion, why have we failed to meet the set targets? ***(****Probe – While implementing any of the strategies, what have been the challenges?)*  What more needs to be done to increase the proportion of health facility deliveries? |
| ***Human Resource /Work Environment Issues***  One of the strategies was to train, recruit and deploy skilled providers at health centres and hospitals. What specific strategies has Ministry of Health implemented to recruit and deploy skilled birth attendants in the facilities?  In the official documents (eg Roadmap for maternal child health), the priority was the recruitment of midwives at health centre II and III. To what extent has the recruitment of midwives been implemented?  Another strategy was the training of comprehensive nurse midwives who were expected to take on both nursing and midwifery roles. To what extent has this strategy been implemented?  Another strategy was incentive schemes for attraction and retention of health workers particularly for hard to reach areas. What has been done, and what are the challenges with the implementation of this strategy?  Another strategy was to provide appropriate physical infrastructure for emergency obstetric care up to Health centre II. What has been done, and what are the challenges with the implementation of this strategy?  Another strategy was to provide appropriate essential supplies and logistics for emergency obstetric care up to Health centre II. What has been done, and what are the challenges with the implementation of this strategy?  To what extent have statutory reforms been done to reflect current on the ground realities for example: a) to allow midwives to provide some aspects of basic emergency obstetric care such as removing retained placenta? b) to allow clinical officers to conduct normal deliveries?  ***(Probe for information on implementation of the Scope of practice, guidelines and standards of care, code of conduct etc)***  Do you have any other reflections on what we have discussed? |

***Thank you for your time.***
